# Supplementary material for: Serum Metabolome Adaptations Following 12 Weeks of High-Intensity Interval Training or Moderate-Intensity Continuous Training in Obese Older Adults
Source: Metabolites. 2023 Jan 29;13(2):198. doi: 10.3390/metabo13020198 (PMC9967246; doi:10.3390/metabo13020198)
Supplement: Supplementary file 1 [file metabolites-13-00198-s001.zip › Supplementary_HIIT_MICT_Revised.pdf]

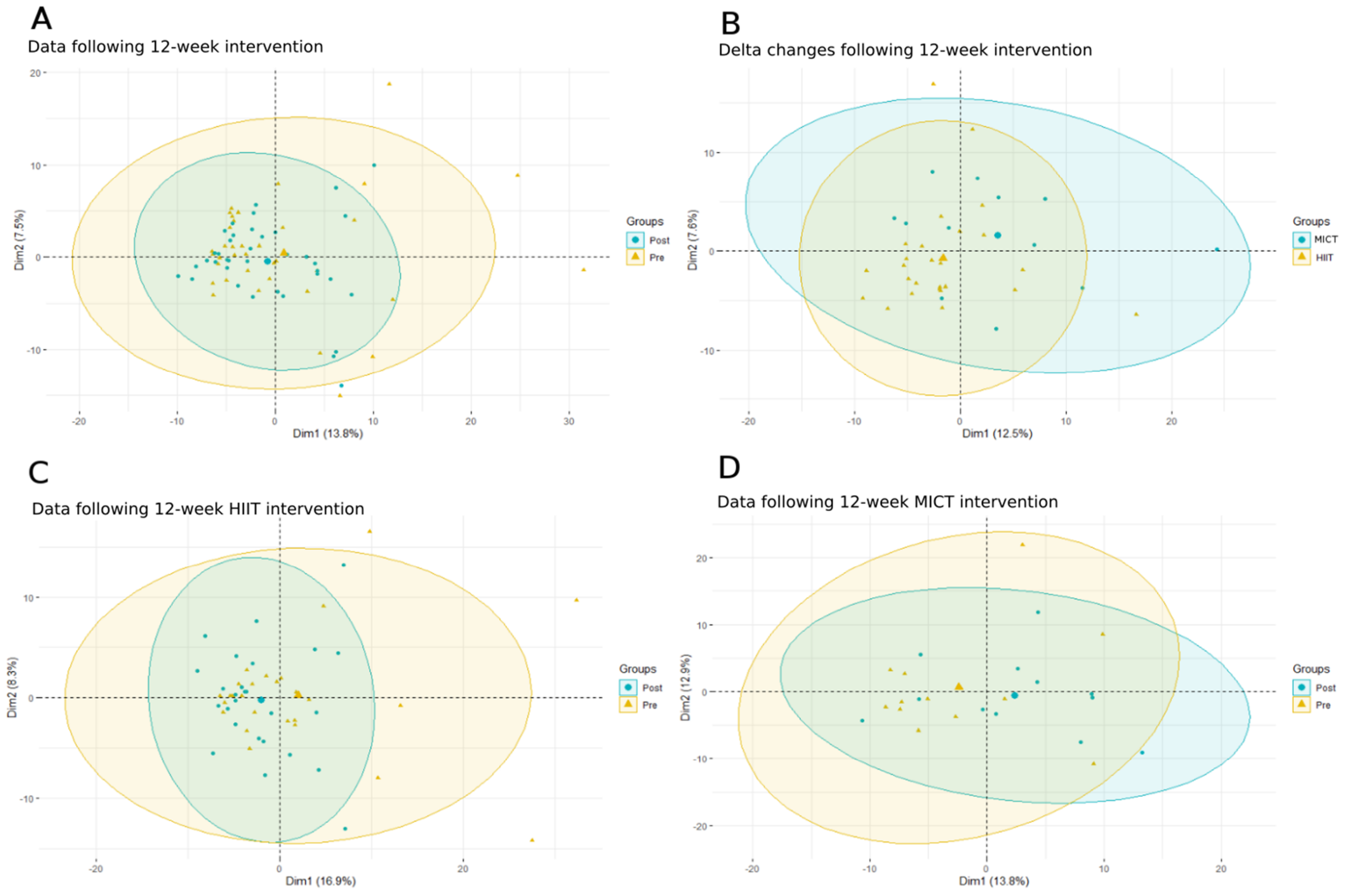

Figure S1: Principal Component Analysis (PCA) of the metabolome following a 12-week HIIT or MICT in obese older adults. PCA plots performed following the overall 12-week HIIT and MICT interventions together (A); on the delta changes following the 12-week HIIT and MICT interventions (B) following the 12-week HIIT intervention alone (C), following the 12-week MICT intervention alone (D). Individuals refers to participants; Pre = before the 12-week intervention; Post = after the 12-week

Table S1: Effect of a 12−week High-Intensity Interval Training (HIIT) and Moderate-Intensity Continuous Training (MICT) on functional capacities and skeletal muscle function parameters in obese older adults.

Parameters	HIIT (n = 26)		MICT (n = 12)		*p*−value	
	Pre	Post	Pre	Post	Time effect	Time×Group effect
**Functional capacities**						
6 min walking test (m)	548.61 ± 80.75	617.81 ± 93.05 ###	534.08 ± 90.15	553.17 ± 75.81	**< 0.0001**	**0.02**
Step test (n)	28.76 ± 4.35	33.04 ± 5.33 ###	24.08 ± 7.14	25.25 ± 5.54	**< 0.0001**	**0.005**
4 m walk test normal (m/s)	1.37 ± 0.16	1.47 ± 0.15 ###	1.24 ± 0.18	1.34 ± 0.22 ##	**< 0.0001**	0.98
4 m walk test fast (m/s)	1.93 ± 0.22	2.07 ± 0.25	1.80 ± 0.33	2.01 ± 0.73	**0.02**	0.60
Unipodal Balance Test (s/60s)	24.73 ± 16.31	37.17 ± 20.42 ###	32.55 ± 21.94	40.01 ± 25.93	**0.0002**	0.39
Chair test (s)	20.19 ± 5.33	16.19 ± 4.15 ###	23.91 ± 6.19	22.37 ± 4.71	**0.0001**	0.13
Timed Up and Go test (s)	10.20 ± 1.35	9.13 ± 1.12 ##	10.91 ± 2.21	9.73 ± 2.89 #	**0.0004**	0.86
**Skeletal muscle function**						
Handgrip strength (kg)	34.01 ± 12.58	33.98 ± 11.07	33.28 ± 13.75	25.54 ± 16.95 #	0.23	0.09
Handgrip strength/BW	0.41 ± 0.12	0.41 ± 0.10	0.42 ± 0.14	0.39 ± 0.14	0.73	0.49
Handgrip strength/ALM	6.72 ± 3.36	6.17 ± 0.84	6.27 ± 1.18	6.14 ± 1.58	0.55	0.55
Quadriceps strength (N)	407.11 ± 191.90	386.55 ± 157.29	482.03 ± 185.60	499.16 ± 197.30	0.65	0.07
Quadriceps/BW	4.96 ± 1.97	4.77 ± 1.47	5.99 ± 1.78	6.18 ± 1.90	0.45	0.09
Quadriceps/LLM	15.82 ± 11.16	13.95 ± 7.93 #	27.84 ± 7.08	29.27 ± 7.61	0.44	**0.02**
Lower limb power (W)	162.08 ± 77.76	185.35 ± 77.70	120.00 ± 66.13	176.42 ± 77.81	**0.0001**	0.05

Data are presented as: mean ± SD. HIIT = high−intensity interval training; MICT = moderate−intensity continuous training; Pre = before the 12−week intervention; Post = after the 12−week intervention; BW = body weight; ALM = arms lean mass; LLM = legs lean lass; Quad = quadriceps. Time effect and Time×Group effects were analyzed using two−way repeated measures ANOVA. # $p < 0.05$, ## $p < 0.01$, ### $p < 0.001$ = Significant intra−group differences between pre and post intervention using two−ways repeated measures ANOVA followed by post−hoc analyses done with simultaneous tests for general linear hypotheses.

Table S2: Effect of a 12−week High−Intensity Interval Training (HIIT) and Moderate−Intensity Continuous Training (MICT) on body composition parameters in obese older adults.

Parameters	HIIT (n = 26)		MICT (n = 12)		*p*−value	
	**Pre**	**Post**	**Pre**	**Post**	**Time effect**	**Time×Group effect**
**Anthropometry**						
Body Weight (kg)	82.11 ± 14.39	81.98 ± 14.22	79.86 ± 16.99	80.52 ± 16.93	0.82	0.49
BMI (kg/m²)	30.28 ± 5.38	30.24 ± 5.36	29.09 ± 5.85	29.34 ± 5.73	0.81	0.47
**Fat and lean mass (DXA)**						
Total lean mass (kg)	47.65 ± 9.33	48.36 ± 9.72	50.25 ± 12.92	47.89 ± 12.98	0.06	0.41
Arms lean mass (kg)	5.54 ± 1.75	5.60 ± 1.67	5.27 ± 1.76	5.24 ± 1.80	0.64	0.95
Legs lean mass (kg)	16.88 ± 3.47	17.21 ± 3.64 [#]	17.02 ± 4.69	16.86 ± 4.38	0.11	**0.04**
Total fat mass (%)	37.75 ± 7.93	37.27 ± 7.67	37.34 ± 7.22	37.02 ± 7.04	0.10	0.76
Arms fat mass (%)	35.94 ± 10.76	35.14 ± 10.33	32.47 ± 10.38	34.12 ± 10.67 [#]	0.97	**0.01**
Legs fat mass (%)	35.79 ± 11.11	35.11 ± 11.01	34.81 ± 11.08	33.92 ± 10.61	**0.02**	0.77
Android fat mass (%)	47.07 ± 7.45	46.65 ± 7.55	47.47 ± 5.90	47.54 ± 5.66	0.47	0.54
Gynoid fat mass (%)	39.88 ± 10.48	39.59 ± 10.30	38.75 ± 11.05	38.80 ± 10.77	0.54	0.59
**Muscle composition (pQCT)**						
Total muscle area (cm²)	103.66 ± 29.40	102.53 ± 32.17	104.22 ± 29.82	100.64 ± 22.77	0.25	0.41
Total fat area (cm²)	91.65 ± 52.16	86.72 ± 48.20	76.69 ± 47.61	74.80 ± 47.46	0.23	0.66
Subcutaneous fat area (cm²)	86.59 ± 51.85	81.90 ± 47.46	72.16 ± 47.38	70.02 ± 47.24	0.25	0.73
Intramuscular fat area (cm²)	5.05 ± 2.31	4.83 ± 2.35	4.65 ± 2.54	4.36 ± 1.89	0.65	0.95

Data are presented as: mean ± SD. HIIT = high−intensity interval training; MICT = moderate−intensity continuous training; Pre = before the 12−week intervention; Post = after the 12−week intervention; DXA = dual−energy X−ray absorptiometry; pQCT = peripheral quantitative computed tomography; BMI = body mass index. Time effect and Time×Group effect were analyzed using two−way repeated measures ANOVA. [#] $p < 0.05$ = Significant intra−group differences between pre and post intervention using two−ways repeated measures ANOVA followed by post−hoc analyses done with simultaneous tests for general linear hypotheses.

Table S3: Effect of a 12−week High−Intensity Interval Training (HIIT) and Moderate−Intensity Continuous Training (MICT) on biological parameters in obese older adults.

Parameters	HIIT (n = 26)		MICT (n = 12)		*p*−value	
	Pre	Post	Pre	Post	Time effect	Time×Group effect
**Biological parameters**						
Adiponectin (µg.ml^{-1})	14.51 ± 7.98	14.24 ± 8.04	12.03 ± 3.66	12.33 ± 4.80	0.87	0.61
Leptin (ng.ml^{-1})	22.72 ± 18.82	26.23 ± 21.41	24.27 ± 21.90	23.35 ± 18.54	0.31	0.31
Adiponectin/leptin	0.85 ± 0.85	1.21 ± 1.60	1.21 ± 1.60	1.00 ± 1.04	0.29	0.59
Free fatty acids (mmol.l^{-1})	0.52 ± 0.15	0.45 ± 0.18	0.53 ± 0.19	0.54 ± 0.21	0.19	0.27
Total cholesterol (mmol.l^{-1})	4.97 ± 1.12	5.00 ± 1.07	4.77 ± 0.78	4.93 ± 0.74	0.56	0.63
HDL (mmol.l^{-1})	1.43 ± 0.37	1.46 ± 0.32	1.32 ± 0.30	1.35 ± 0.35	0.08	0.90
LDL (mmol.l^{-1})	2.85 ± 0.92	2.92 ± 0.89	2.73 ± 0.71	2.87 ± 0.61	0.37	0.79
Triglycerides (mmol)	1.53 ± 0.71	1.35 ± 0.68	1.59 ± 0.55	1.54 ± 0.69	0.09	0.48
Ferritin (µg.l^{-1})	138.33 ± 91.43	122.81 ± 94.46 $^{\#}$	93.65 ± 48.65	82.67 ± 43.54	**0.01**	0.68
IGF1 (µg.ml^{-1})	0.08 ± 0.02	0.09 ± 0.02	0.09 ± 0.02	0.09 ± 0.03	0.16	0.77
IGFBP3 (µg.ml^{-1})	1.78 ± 0.39	1.87 ± 0.34	1.73 ± 0.25	1.70 ± 0.27	0.22	0.16
IGF1/IGFBP3	0.05 ± 0.01	0.05 ± 0.01	0.05 ± 0.01	0.05 ± 0.01	0.45	0.23
Glucose (mmol.l^{-1})	5.99 ± 1.28	6.04 ± 1.46	5.70 ± 0.47	5.70 ± 0.80	0.72	0.81
Insulin (pmol)	49.06 ± 23.57	49.03 ± 29.36	55.10 ± 35.51	46.41 ± 26.04	0.29	0.13
QUICKI	0.42 ± 0.05	0.43 ± 0.05	0.42 ± 0.04	0.43 ± 0.04	0.14	0.64
HOMA−IR (M.U)	2.19 ± 1.19	2.21 ± 1.43	2.35 ± 1.57	1.94 ± 1.05	0.37	0.16

Data are presented as: mean ± SD. HIIT = High−Intensity Interval Training; MICT = moderate−intensity continuous training; Pre = before the 12−week intervention; Post = after the 12−week intervention; HDL = high−density lipoprotein; LDL = low−density lipoprotein; IGF−1 = insulin−like growth factor−1; IGFBP−3 = insulin−like growth factor binding protein−3; QUICKI = quantitative insulin−sensitivity check index; HOMA = homeostatic model assessment for insulin resistance; M.U = mass unit. Time effect and Time×Group effect were analyzed using two−way repeated measures ANOVA. $^{\#}$ $p < 0.05$ = Significant intra−group differences between pre and post intervention using two−ways repeated measures ANOVA followed by post−hoc analyses done with simultaneous tests for general linear hypotheses.

Table S4−a: False Discovery Rate Analysis (FDR) on the 50 significant metabolites obtained following a 12−week High−Intensity Interval Training (HIIT) or Moderate−Intensity Continuous Training (MICT) in obese older adults.

**Metabolites**	**ANOVA**		**FDR (BH)**	
	**Time effect**	**Time×Training effect**	**Time effect**	**Time×Training effect**
TCA cycle				
2−oxoglutaric acid	**0.0002****	0.13	**0.03**	0.57
3−methylhistidine	**0.006****	0.79	0.35	0.96
Aspartic acid	0.12	**0.005****	0.63	0.17
Fumaric acid	**0.001****	0.71	0.09	0.94
Pyruvic acid and Oxaloacetic acid	**0.03***	0.56	0.56	0.89
Aspartate/Malate	0.13	**0.003****	0.65	0.15
Carbohydrate metabolism				
Acetic acid	**0.04***	0.32	0.56	0.17
Glyceric acid	**<0.0001*****	0.08	**0.03**	0.17
Ribitol	0.75	**0.04***	0.51	0.51
Xylitol	**0.02***	**0.004****	0.15	0.15
Xylose	0.31	**0.008****	0.73	0.25
Amino acid metabolism				
2−aminoadipic acid	**0.03***	0.08	0.52	0.52
2−hydroxybutyric acid	0.12	**0.04***	0.63	0.58
2−oxovaleric acid	**0.01***	0.57	0.35	0.89
3−hydroxybutyric acid	**0.03***	0.44	0.52	0.81
Ketoisovaleric acid	**0.002****	0.05	0.14	0.52
Hypotaurine	0.07	**0.04***	0.57	0.52
Inosine	**0.01***	**0.01***	0.35	0.23
Ornithine	**0.01***	0.21	0.35	0.65
Proline	0.68	**0.04***	0.92	0.52
Uric acid	0.36	**0.002****	0.74	0.15
Xanthine	0.27	**0.01***	0.71	0.23

FDR = False Discovery Rate, BH = Benjamini−Hochberg, TCA = Tricarboxylic acid, / = ratio, *: < 0.05, **: < 0.01, ***: < 0.001

Table S4−b: False Discovery Rate Analysis (FDR) on the 50 significant metabolites obtained following a 12−week High−Intensity Interval Training (HIIT) or Moderate−Intensity Continuous Training (MICT) in obese older adults.

**Metabolites**	**ANOVA**		**FDR (BH)**	
	**Time effect**	**Time×Training effect**	**Time effect**	**Time×Training effect**
Fat metabolism				
Acetylcarnitine	**0.02***	0.71	0.49	0.94
Arachidonic acid	**0.02***	0.78	0.52	0.96
Butanoic acid	**0.03***	0.84	0.52	0.97
Carnitine C18:0	**0.04***	0.55	0.56	0.88
Ceramide (18:1/22:0)	0.94	**0.02***	0.98	0.71
Ceramide (18:1/24:0)	0.39	**0.03***	0.74	0.49
DG (18:1/18:3)	0.86	**0.0004*****	0.96	0.14
DG (20:4/18:2)	**0.04***	0.67	0.56	0.94
Isobutyric acid	**0.007****	0.15	0.35	0.57
Linoleic acid	**0.03***	0.66	0.69	0.52
Margaric acid	**0.001****	0.28	0.09	0.72
Pantothenic acid	0.09	**0.003****	0.57	0.15
PCae (15:0)	0.18	**0.01***	0.68	0.25
PCae (16:0)	0.19	**0.02***	0.69	0.41
PCae (20:2)	0.75	**0.03***	0.93	0.49
PCae (22:1)	0.29	**0.03***	0.72	0.80
PCae (22:4)	0.92	**0.04***	0.52	0.52
PEaa (36:1)	0.67	**0.04***	0.92	0.51
PEaa (38:6)	0.74	**0.02***	0.93	0.41
PEee (19:1)	**0.02***	**0.03***	0.49	0.49
TG (12:0/12:0/16:1)	**0.04***	0.48	0.56	0.82
TG (12:0/14:0/16:0)	**0.02***	0.24	0.49	0.70
TG (14:0/16:0/16:0)	**0.04***	0.74	0.56	0.94
TG (14:0/16:2/16:2)	**0.03**	0.81	0.52	0.97
TG (16:1/18:1/18:0)	0.35	**0.003****	0.15	0.57
TG (16:1/18:3/18:2)	0.09	**0.004****	0.74	0.15
TG (16:1/18:3/20:4)	0.96	**0.02***	0.98	0.41
TG (16:2/18:2/18:2)	**0.04***	**0.002****	0.56	0.15
Undecanoic acid	**0.06***	**0.001****	0.57	0.15

FDR = False Discovery Rate, BH = Benjamini−Hochberg, DG = Diglyceride; PCae = acyl−alkyl−phosphatidylcholine; PEaa = alkyl acyl−phosphatidylethanolamine; PEee = ether−phosphatidylethanolamine; TG = Triglyceride *: < 0.05, **: < 0.01, ***: < 0.001

Table S5−a: False Discovery Rate Analysis (FDR) for each type of intervention alone on the 50 significant metabolites obtained following a 12−week High−Intensity Interval Training (HIIT) or Moderate−Intensity Continuous Training (MICT) in obese older adults.

**Metabolites**	**ANOVA**		**FDR (BH)**	
	**HIIT effect**	**MICT effect**	**HIIT effect**	**MICT effect**
TCA cycle				
2−oxoglutaric acid	**0.01***	**0.0007*****	0.29	**0.04***
3−methylhistidine	**0.03***	0.06	0.94	0.14
Aspartic acid	**0.004****	0.12	0.19	0.65
Fumaric acid	**0.003****	0.09	0.17	0.59
Pyruvic acid and Oxaloacetic acid	0.14	0.09	0.58	0.59
Aspartate/Malate	**0.003****	0.08	0.17	0.58
Carbohydrate metabolism				
Acetic acid	**0.02***	0.72	0.42	0.72
Glyceric acid	**< 0.0001*****	0.19	**0.003****	0.69
Ribitol	0.15	0.13	0.58	0.65
Xylitol	0.79	**0.0003*****	0.93	**0.04***
Xylose	0.47	**0.005****	0.72	0.14
Amino acid metabolism				
2−aminoadipic acid	0.39	**0.008****	0.66	0.17
2−hydroxybutyric acid	**0.01***	0.38	0.38	0.56
2−oxovaleric acid	**0.01***	0.29	0.29	0.82
3−hydroxybutyric acid	0.14	0.05	0.58	0.56
Ketoisovaleric acid	0.12	**0.001****	0.55	**0.04***
Hypotaurine	0.74	**0.007****	0.89	0.14
Inosine	**0.0008*****	0.48	0.09	0.86
Ornithine	0.17	**0.01****	0.59	0.19
Proline	0.41	0.06	0.58	0.56
Uric acid	0.25	**0.002****	0.59	**0.04***
Xanthine	0.54	**0.005****	0.77	0.14

FDR = False Discovery Rate, BH = Benjamini−Hochberg, HIIT = High−Intensity Interval Training; MICT = Moderate−Intensity Continuous Training; TCA = Tricarboxylic acid, / = ratio, *: < 0.05, **: < 0.01, ***: < 0.001

Table S5−b: False Discovery Rate Analysis (FDR) for each type of intervention alone on the 50 significant metabolites obtained following a 12−week High−Intensity Interval Training (HIIT) or Moderate−Intensity Continuous Training (MICT) in obese older adults.

**Metabolites**	**ANOVA**		**FDR (BH)**	
	**HIIT effect**	**MICT effect**	**HIIT effect**	**MICT effect**
Fat metabolism				
Acetylcarnitine	**0.03***	0.28	0.44	0.82
Arachidonic acid	0.08	0.12	0.53	0.65
Butanoic acid	0.09	0.15	0.53	0.65
Carnitine C18:0	**0.04***	0.37	0.44	0.82
Ceramide (18:1/22:0)	0.10	0.06	0.65	0.56
Ceramide (18:1/24:0)	0.05	0.16	0.44	0.65
DG (18:1/18:3)	**0.04***	**0.001****	0.44	**0.04**
DG (20:4/18:2)	0.05	0.39	0.44	0.83
Isobutyric acid	0.70	**0.002****	0.17	0.90
Linoleic acid	0.03	0.33	0.38	0.36
Margaric acid	**0.03***	0.006	0.44	0.14
Pantothenic acid	0.71	**0.0007*****	0.88	**0.04**
PCae (15:0)	**0.01***	0.15	0.29	0.65
PCae (16:0)	**0.02***	0.22	0.29	0.74
PCae (20:2)	0.13	0.10	0.45	0.62
PCae (22:1)	**0.04***	0.22	0.88	0.90
PCae (22:4)	0.25	0.06	0.45	0.56
PEaa (36:1)	0.12	0.12	0.55	0.65
PEaa (38:6)	0.10	0.07	0.55	0.58
PEee (19:1)	0.48	**0.003****	0.73	0.10
TG (12:0/12:0/16:1)	0.18	0.08	0.59	0.59
TG (12:0/14:0/16:0)	0.17	0.03	0.59	0.49
TG(14:0/16:0/16:0)	0.12	0.14	0.55	0.65
TG (14:0/16:2/16:2)	0.09	0.15	0.59	0.65
TG (16:1/18:1/18:0)	0.33	**0.003****	0.65	0.10
TG (16:1/18:3/18:2)	0.83	0.0009	0.44	**0.04**
TG (16:1/18:3/20:4)	0.16	0.05	0.94	0.55
TG (16:2/18:2/18:2)	0.98	**0.0003*****	0.98	**0.04**
Undecanoic acid	**0.0008*****	0.06	0.09	0.56

FDR = False Discovery Rate, BH = Benjamini−Hochberg, HIIT = High−Intensity Interval Training; MICT = Moderate−Intensity Continuous Training; DG = Diglyceride; PCae = acyl−alkyl−phosphatidylcholine; PEaa = alkyl acyl−phosphatidylethanolamine; PEee = ether−phosphatidylethanolamine; TG = Triglyceride *: < 0.05, **: < 0.01, ***: < 0.001

List of inclusion and exclusion criteria of the participants

To be included in this study, participants had to meet the following criteria: 1) age 60 and over; 2) obese (BMI between 30 and 40 $kg.m^{-2}$ or fat mass (%; DXA) equal or superior to 27% in men and 40% in women) or a waist circumference greater than 102 cm for men and 88 cm for women; 3) inactive (less than two hours of structured physical activity per week); 4) no involvement in a vigorous exercise program for at least 12 months; 5) able to follow the exercise training; 6) stable weight (± 5 kg) for 6 months; 7) non−smokers and moderate drinkers (max: 15 g/day of alcohol); 8) able to understand French or English; and 9) postmenopausal for women (*i.e.,* 12 consecutive months without menses). Exclusion criteria were the following: 1) presence of metal implant (pacemaker); 2) asthma requiring oral steroid treatment; 3) use of medication that could affect metabolism or cardiovascular function; 4) use of anticoagulants (only for participants undergoing biopsies). Participants with diagnosed but untreated neurological, cardiovascular or lung diseases, or cognitive disorders were also excluded.
